# Short-term outcomes of phosphodiesterase type 5 inhibitors for fetal growth restriction: a study protocol for a systematic review with individual participant data meta-analysis, aggregate meta-analysis, and trial sequential analysis

**DOI:** 10.1186/s13643-021-01849-5

**Published:** 2021-12-03

**Authors:** Jessica Liauw, Katie Groom, Wessel Ganzevoort, Christian Gluud, Christopher J. D. McKinlay, Andrew Sharp, Laura Mackay, Chirag Kariya, Ken Lim, Peter von Dadelszen, Jacqueline Limpens, Janus C. Jakobsen, the STRIDER Consortium, Francois Audibert, Zarko Alfirevic, Philip Baker, Emmanuel Bujold, Youkee Chung, Christine Cornforth, Wessel Ganzevoort, Sanne J. Gordijn, Katie Groom, Christian Gluud, Janus C. Jakobsen, Edward D. Johnstone, Chirag Kariya, Louise Kenny, Tang Lee, Larry Li, Jessica Liauw, Ken Lim, Laura Magee, Laura Mackay, Lesley McCowan, Chris McKinlay, Ben W. Mol, Wes Onland, Aris Papageorghiou, Anouk Pels, Andrew Sharp, Peter von Dadelszen

**Affiliations:** 1 Department of Obstetrics and Gynaecology, University of British Columbia, Room C420-4500 Oak Street, Vancouver, British Columbia V6H 3N1 Canada; 2 Liggins Institute, University of Auckland, Auckland, New Zealand; 3 Amsterdam University Medical Centers, University of Amsterdam, Amsterdam, The Netherlands; 4 Copenhagen Trial Unit, Centre for Clinical Intervention Research, Capital Region of Denmark, Rigshospitalet, Copenhagen University Hospital, Copenhagen, Denmark; 5 Harris-Wellbeing Preterm Birth Centre, University of Liverpool, Liverpool, UK; 6 BC Cancer, Vancouver, British Columbia Canada; 7 King’s College, London, UK; 8 Department of Regional Health Research, The Faculty of Heath Sciences, University of Southern Denmark, Odense, Denmark

**Keywords:** Fetal growth restriction, Phosphodiesterase type 5 inhibitor, Randomised clinical trial, Trial sequential analysis, Aggregate meta-analysis, Individual patient data meta-analysis

## Abstract

**Abstract:**

**Background:**

Early onset fetal growth restriction secondary to placental insufficiency can lead to severe maternal and neonatal morbidity and mortality. Pre-clinical studies and a few small randomised clinical trials have suggested that phosphodiesterase type 5 (PDE-5) inhibitors may have protective effects against placental insufficiency in this context; however, robust evidence is lacking. The STRIDER Consortium conducted four randomised trials to investigate the use of a PDE-5 inhibitor, sildenafil, for the treatment of early onset fetal growth restriction. We present a protocol for the pre-planned systematic review with individual participant data meta-analysis, aggregate meta-analysis, and trial sequential analysis of these and other eligible trials. The main objective of this study will be to evaluate the effects of PDE-5 inhibitors on neonatal morbidity compared with placebo or no intervention among pregnancies with fetal growth restriction.

**Methods:**

We will search the following electronic databases with no language or date restrictions: OVID MEDLINE, OVID EMBASE, the Cochrane Controlled Register of Trials (CENTRAL), and the clinical trial registers Clinicaltrials.gov and World Health Organisation International Clinical Trials Registry Platform (ICTRP). We will identify randomised trials of PDE-5 inhibitors in singleton pregnancies with growth restriction. Two reviewers will independently screen all citations, full-text articles, and abstract data. Our primary outcome will be infant survival without evidence of serious adverse neonatal outcome. Secondary outcomes will include gestational age at birth and birth weight *z*-scores. We will assess bias using the Cochrane Risk of Bias 2 tool. We will conduct aggregate meta-analysis using fixed and random effects models, Trial Sequential Analysis, and individual participant data meta-analysis using one- and two-stage approaches. The certainty of evidence will be assessed with GRADE.

**Discussion:**

This pre-defined protocol will minimise bias during analysis and interpretation of results, toward the goal of providing robust evidence regarding the use of PDE-5 inhibitors for the treatment of early onset fetal growth restriction.

**Systematic review registration:**

PROSPERO (CRD42017069688).

**Supplementary Information:**

The online version contains supplementary material available at 10.1186/s13643-021-01849-5.

## Background

Early onset fetal growth restriction, considered as failure of a fetus to reach its full growth potential diagnosed at less than 32 weeks’ gestation [1], is associated with stillbirth, preterm birth, neonatal and childhood long-term morbidity and mortality, and maternal hypertensive disorders of pregnancy [2–6]. The most common cause of early onset fetal growth restriction is uteroplacental insufficiency, secondary to inadequate remodelling of maternal spiral arteries with subsequent reduced blood flow to the placental bed leading to hypoxic-ischaemic injury [7].

Pre-clinical studies have suggested that phosphodiesterase type 5 (PDE-5) inhibitors may ameliorate fetal growth restriction by promoting vasodilatation and blood flow in the uteroplacental circulation [8–11]. PDE-5 enzymes degrade cyclic guanosine monophosphate (cGMP), a nucleotide implicated in the activation of nitric oxide, a potent vasodilator. Animal models have suggested multiple mechanisms by which PDE-5 inhibitors may have protective effects in the setting of uteroplacental insufficiency [12]. In two small randomised clinical trials, the use of PDE-5 inhibitors was associated with improved umbilical, middle cerebral, and uterine artery Doppler waveform indices in pregnancies affected by fetal growth restriction [13, 14]. In a further randomised clinical trial in women with preeclampsia, PDE-5 inhibitor use was associated with prolongation of pregnancy by 4 days when compared with placebo [15], while another randomised clinical trial showed no effect on pregnancy prolongation [16].

These pre-clinical studies, animal models, and small randomised clinical trials supported the need for larger-scale randomised clinical trials to investigate the utility of PDE-5 inhibitors for the treatment of fetal growth restriction. The STRIDER (Sildenafil TheRapy In Dismal prognosis Early-onset fetal growth Restriction) Consortium was established in 2012 [17] to further investigate the potential of PDE-5 inhibitors in this setting. This multinational collaboration established four randomised clinical trials in the UK, New Zealand/Australia, the Netherlands, and Canada. An individual participant data meta-analysis was prospectively planned by the Consortium [18] prior to initiation of the individual trials, to enable further examination of clinically significant outcomes and to allow more meaningful subgroup analysis. The results of three STRIDER trials have been published to date [19–21]. These trials demonstrated no beneficial effects of sildenafil versus placebo on fetal growth velocity [19], prolongation of pregnancy [20], or perinatal mortality/neonatal morbidity [21]. The Dutch STRIDER trial [21] raised concerns about an increased risk of neonatal pulmonary hypertension in the sildenafil group; for this reason and the low likelihood of showing benefit, the Dutch and Canadian STRIDER trials were stopped before the recruitment targets were achieved [22]. A recent systematic review with meta-analysis [23] summarised randomised trials on the effect of PDE-5 inhibitors on maternal and fetal outcomes, and found an increase in birthweight in pregnancies in the intervention group. However, this review noted high heterogeneity between the included studies (*I*^2^ = 96%) and, because the search terminated in September 2018, it did not include published results from all the STRIDER trials.

Here, we present a protocol for an individual participant data meta-analysis to answer the question: what are the short-term (perinatal, neonatal, and maternal) effects of PDE-5 inhibitors, compared to placebo or no treatment, when used in the treatment of fetal growth restriction? Focusing on a specific indication and using individual data from all the STRIDER and other recent trials will enable us to address the limitations in the prior review [23] and may provide definitive evidence on the safety and effectiveness of PDE-5 inhibitors in this context.

## Methods

We will conduct the systematic review according to this protocol and report any deviations from it in the published review under ‘Differences between protocol and review’. This protocol was initially registered in PROSPERO (# CRD42017069688) and amended in October 2020 to reflect refinements to the outcome, risk of bias assessment, and analysis plan (see “Discussion” section for further details). We will report this review in accordance with the PRISMA Extension for systematic reviews and meta-analysis of individual participant data [24] (PRISMA-IPD, see checklist in Additional file 1).

Separate ethics approval for this study is not required as we intend to use data from the STRIDER trials and other trials. Each trial was approved by local ethics boards; for STRIDER trials these approvals included use of the data in this pre-planned individual participant data meta-analysis.

### Identification of eligible trials

#### Search strategy

We will search the following electronic databases from 1946 to September 11, 2020 initially (see Additional file 2), with no language or date restrictions: OVID MEDLINE, OVID EMBASE, the Cochrane Controlled Register of Trials (CENTRAL) and the clinical trial registers Clinicaltrials.gov and WHO-ICTRP. We will include conference abstracts published in these databases. We will not hand search conference proceedings. Just prior to the submission of our paper we will conduct an updated literature search.

The search will consist of text words and database specific controlled terms (i.e., MeSH-terms in MEDLINE) for fetal growth restriction (or conditions which may relate to fetal growth restriction, i.e., preeclampsia) and PDE-5 inhibitors. This search will be combined with a search filter to retrieve human randomised clinical trials. We will cross-check the reference lists and the cited articles (via Web of Science) of relevant papers for additional relevant trials. The retrieved records will be imported in ENDNOTE and duplicates will be removed. A search strategy for MEDLINE is included in Additional file 2.

#### Study selection

We will include data from all four STRIDER Consortium trials, which are housed by the Perinatal Clinical Trial Unit at the University of British Columbia, Canada. Two investigators will review all citations retrieved by the search strategy and select all trials, which meet our inclusion criteria. Discrepancies will be resolved by discussion and, if required, a third investigator.

#### Inclusion criteria at the study level

##### Types of studies

- Randomised clinical trials assessing the effects of PDE-5 inhibitors in women with pregnancies with fetal growth restriction. Only studies that include fetal growth restriction as an inclusion criterion for their trial population will be included (as defined by trialists). Trials co-administering other interventions that may impact pregnancy outcomes in the context fetal growth restriction will be included, as long as these interventions are planned to be equally administered to intervention and control groups.

##### Participants

Studies which include women with singleton pregnancies affected by fetal growth restriction (as defined by individual trials).

##### Types of intervention

Any PDE-5 inhibitor (e.g. sildenafil, tadalafil) at any dose and by any route of administration with the intention of multiple dose administration since single dose regimens may have different characteristics and mechanisms of action.

##### Types of control

Placebo or no intervention.

#### Inclusion criteria at the individual level

- Participants: women with singleton pregnancies affected by fetal growth restriction (as defined by individual trials), since we are interested in the use of PDE-5 inhibitors for this indication only.

### Data sharing agreement

Authors of eligible trials who agree to participate will be asked to review and sign a Data Sharing Agreement prior to the transfer of individual participant data.

### Primary outcome

#### For the neonate

Infant survival without evidence of serious adverse neonatal outcome.

Serious adverse neonatal outcome, defined as one or more of the following:

- Cerebral intraventricular haemorrhage (Papile grade 3 or 4 [25], or as defined by individual trials)
- Cystic periventricular leukomalacia (grade two or more [26], or as defined by individual trials).
- Bronchopulmonary dysplasia (as defined by individual trials).
- Necrotising enterocolitis requiring surgery (as defined by individual trials).
- Retinopathy of prematurity requiring treatment (as defined by individual trials).

Components of the composite outcome will be assessed separately in the exploratory analysis.

### Secondary outcomes

#### For the neonate

- Gestational age at birth (among liveborn neonates).
- Birth weight z-score (among liveborn neonates) [27].

#### For the woman

- Maternal preeclampsia (as defined by individual trials).

### Exploratory outcomes

#### For the neonate

- Persistent pulmonary hypertension in the neonate (as defined by individual trials).
- Stillbirth.
- Neonatal or infant death.
- Cerebral intraventricular haemorrhage (Papile grade 3 or 4 [25] or as defined by individuals trials).
- Cystic periventricular leukomalacia (grade 2 or more [26], or as defined by individual trials).
- Bronchopulmonary dysplasia (as defined by individual trials).
- Necrotising enterocolitis requiring surgery (as defined by individual trials).
- Retinopathy of prematurity requiring treatment (as defined by individual trials).
- Persistent pulmonary hypertension as defined by the Consortium (after completion of the trials).
- Use of nitric oxide (neonatal).
- Fetal growth velocity post-treatment: [19]
  - Post-treatment growth velocity will be calculated from Z-scores at the recruitment and day 14 assessments. Where delivery or fetal death occurred before the day 14 assessment, the longest interval available will be used (i.e. to a minimum of 48 h).
- Birthweight (grams).
- Pregnancy prolongation (in days).

#### For the woman

- Maternal systolic blood pressure 48–72 h after commencing treatment.
- Maternal diastolic blood pressure 48–72 h after commencing treatment.
- Mode of birth: vaginal compared to caesarean section.
- Abnormal maternal serum placental growth factor (PLGF) (as defined by individual trials).

### Assessment time points

All outcomes will be assessed to the time of maximum follow-up as defined by individual trials within 1 year from randomisation.

### Risk of bias assessment

Two authors will independently assess the risk of bias of included trials using the Cochrane Risk of Bias tool (RoB 2) for randomised clinical trials [28]. We will evaluate the methodology of each trial with respect to the five domains outlined by this tool for each outcome, including the risk of bias arising from the randomisation process, risk of bias due to deviations from the intended interventions, risk of bias due to missing outcome data, risk of bias in measurement of the outcome, risk of bias in selection of the reported result, and overall risk of bias. We will determine if, in each domain, there are low, high, or some concerns for risk of bias. We will also check individual participant data for the pattern of treatment allocation, to check randomisation and assess for unusual patterns in treatment allocation [29]. Individual participant data will also be checked for attrition and completeness of outcome data regardless of whether it was reported in the original trial [29]. Trials will be judged at overall low risk of bias if assessed as low risk in all domains of the RoB 2 tool and if no major issues are noted on checking of IPD data. Trials will be judged to be at overall high risk of bias if assessed as high risk in at least one domain, or some concerns for two or more domains, or there are concerns with IPD checking which lowers confidence in the result. During data synthesis, a subgroup analysis will be performed to assess the primary outcome among trials with a high risk of bias compared with those with a low risk of bias (see “Data synthesis” section below).

### Cumulative evidence assessment

The Grading of Recommendations, Assessment, and Development and Evaluation (GRADE) framework [30] will be used to summarise the certainty of evidence for all outcomes. Summary of findings tables will be produced, commenting on the risk of bias, imprecision, inconsistency, indirectness, and publication bias [31].

If there are diverging views regarding risk of bias or assessment of the quality of the evidence, a third author will be involved to achieve consensus.

### Data collection and management

#### Aggregate data

Aggregate data will be extracted independently by two members of the research team. We will extract data on trial eligibility criteria, study methods, participant characteristics, intervention details, outcomes assessed, and primary results for the effects of PDE-5 inhibitors on fetal growth. The data extraction form will be piloted, and discrepancies in data extraction will be discussed with a third member of the research team. Data extraction training will occur before and throughout the data extraction process. The extracted data will be summarised in a table (see “Data synthesis” section, below).

#### Individual participant data

Principal investigators or the primary contact listed on a trial publication or trial registry of eligible studies will be invited by email to collaborate and contribute individual participant data. We will send two follow up emails 1 month apart (for a total of three email invitations over three months). If no reply is received after these three emails, the individual participant data from this study will be considered unattainable.

If a trial author agrees to participate, a data sharing agreement will be signed before any data are transferred. Authors will be asked to provide de-identified data by encrypted, electronic transfer over a secure network. If this is not possible, other arrangements will be made to securely transfer data as needed. Data will be recorded and reformatted for storage in a secure electronic study database. Only study team members will have access to this database. A copy of the received data will be stored prior to reformatting. Reformatted data will be sent back to study authors to verify accuracy prior to analysis.

Individual participant data will be checked for range, internal consistency, extreme values, missing values, and consistency with published reports. Inconsistencies and missing data will be discussed and checked with study authors.

If authors of included trials do not provide individual participant data, these trials will be included in the aggregate meta-analysis only. We will examine the impact of unavailable data in the individual participant data meta-analysis when interpreting our results.

### Individual participant data items

In addition to the outcome data items (see “Primary outcome”, “Secondary outcomes”, and “Exploratory outcomes” sections), we will collect the following baseline characteristics of participants in order to demonstrate any significant differences between the intervention groups. These items were chosen to reflect maternal or pregnancy characteristics which may impact short-term neonatal outcomes. See Additional file 3 for a full list of data variables which will be collected on the individual-participant level.

Maternal characteristics at study entry

- Age.
- Body mass index.
- At least one previous pregnancy ≥20 weeks.
- Ethnicity.
- Smoking status.
- Pre-existing hypertension requiring medication.
- Diabetes (type 1, type 2, and gestational).
- Gestational hypertension.
- Preeclampsia.
- Gestational age at diagnosis of preeclampsia.
- Concurrent treatment with
  - Acetylsalicylic acid (e.g. Aspirin).
  - Low molecular weight heparin.

Pregnancy characteristics at trial entry

- Gestational age (based on ultrasound assessment at < 14 weeks gestation):
  - < 24 weeks + 0 days
  - ≥ 24 weeks + 0 days
  - Gestational age, weeks.
- Abdominal circumference centile [27].
- Estimated fetal weight, g (Hadlock C) [32].
- Umbilical artery:
  - Mean pulsatility index.
  - End diastolic flow absent or reversed.
  - Mean pulsatility index ≤95th centile (normal).
  - Mean pulsatility index >95th centile (abnormal).
- Mean pulsatility index of middle cerebral artery.
- Uterine artery:
  - Mean pulsatility index.
  - Unilateral or bilateral notching.

Pregnancy characteristics after trial entry

- Development of preeclampsia and gestational age at diagnosis of preeclampsia.
- Antenatal corticosteroids administered for fetal lung maturity.
- Magnesium sulphate administered.

### Data synthesis

We will first compare included trials based on key participant characteristics at trial entry (preeclampsia, gestational age, criteria for growth restriction) and intervention details (type, dose, and duration of PDE-5 inhibitor) of each study. This information will be summarised in a table, and will be assessed qualitatively for heterogeneity and eligibility for meta-analysis. We will conduct an aggregate meta-analysis, trial sequential analysis, and an individual participant data meta-analysis.

Binary outcomes will be presented as risk ratios and absolute risk differences with 95% confidence intervals. Continuous outcomes will be presented as differences in means or medians with 95% confidence intervals. For all outcomes, we will also calculate Trial Sequential Analysis-adjusted confidence intervals (see “Trial sequential analysis” section below).

The procedures for each analysis are outlined below:

## 1. Aggregate meta-analysis

We will undertake the meta-analyses according to the *Cochrane Handbook of Systematic Reviews of Interventions* [33], Keus et al. [34], and the eight-step assessment suggested by Jakobsen et al. [35]. We will consider combining all included trials (i.e., those providing individual participant data and trials not providing individual participant data) in the aggregate meta-analysis. We will assess heterogeneity (see “Assessment of Heterogeneity” section below) to judge whether pooling of all trials is warranted and ultimately decide whether meta-analysis should be performed including all trials.

We will use the statistical software Stata version 16 [36] to analyse data (command: meta). We will assess our intervention effects with both a random-effects meta-analysis [37] and fixed-effect meta-analysis for each treatment comparison separately [38]. We will primarily use the more conservative point estimate of the two [35]. The more conservative point estimate is the estimate with the highest *P* value. We will assess a total of four main (primary and secondary) outcomes, and we will therefore consider a *P* value of 0.02 or less as the threshold for statistical significance [35]. We will investigate possible heterogeneity through subgroup analyses. We will use the eight-step procedure to assess if the thresholds for significance are crossed [35]. Where multiple trial arms are reported in a single trial, we will include only the relevant arms. If two comparisons are combined in the same meta-analysis, we will halve the control group to avoid double-counting [33].

### Assessment of heterogeneity

Heterogeneity among trials will be assessed by visual inspection of forest plots. We will calculate the *I*^*2*^ value [33]. If heterogeneity in treatment effect or inconsistency across trials is detected, then the rationale for combining trials will be questioned and the source of heterogeneity will be explored via subgroup analysis.

### Subgroup analysis

We will examine trials in the following subgroups, as specified by the inclusion criteria or published stratified analyses of individual trials:

- Gestational age at inclusion of < 23 weeks and 6 days compared to those with a gestational age at inclusion of 24 weeks or more, since 24 weeks’ gestation is considered a threshold for viability in many jurisdictions [39], so this may influence treatment decisions and outcomes.
- Abdominal circumference at inclusion below the 3rd percentile as defined by individual trials compared to those at or above this threshold, since this is a sole criteria for fetal growth restriction based on international consensus [1], which may influence treatment decisions.
- Estimated fetal weight at inclusion of < 500 g compared to > 500 g.
- Absent or reversed end diastolic flow in the umbilical artery Doppler waveform at inclusion compared to present end diastolic flow at inclusion since this is a proven poor prognostic factor for fetal demise [40].
- Abnormal uterine artery Doppler at inclusion defined as mean pulsatility index (PI) > 95th centile and/or presence of unilateral or bilateral notches at inclusion compared to those with mean PI <95th centile and no notches, since increased resistance in the uterine arteries is associated with placental insufficiency and malperfusion, so it is possible that the intervention may be more effective in these pregnancies [41].
- Abnormal serum placental growth factor (PLGF) at inclusion as defined by individual trials compared to those with normal serum PLGF, since PLGF is an emerging marker for placental insufficiency and malperfusion, so it is possible that the intervention may be more effective in these pregnancies [41].
- Gestational hypertension, preeclampsia, or HELLP syndrome at inclusion as defined by individual trials compared to those without, because previous studies have shown that PDE-5 inhibitors improve outcomes [15] or are not harmful [15, 16], in patients with these conditions.
- STRIDER trials only compared to other trials, as we expect that STRIDER trials may be more homogeneous compared to other trials.
- Trials with a high risk of bias compared to those with a low risk of bias.
- Trials which were industry sponsored compared to those which were not industry sponsored.
- Trials using a daily dose of < 50 mg of PDE-5 inhibitor medication compared to those using a daily dose of > 50 mg.
- Trials using the PDE-5 inhibitor sildenafil compared to those using the PDE-5 inhibitor tadalafil.

Each subgroup analysis will be examined using an interaction test in Stata. We will consider a *P* value of 0.05 or less as indicating a statistically significant difference between subgroups.

### Assessment of reporting bias

If more than 10 trials are included, we will visually inspect funnel plots to assess for reporting bias.

### Dealing with missing data

We will use intention-to-treat data where available. If there are missing data, trial authors will first be contacted to provide the missing data. The impact of missing data will be assessed in our sensitivity analyses, as detailed below.

### Sensitivity analyses for dealing with missing data

For binary outcomes, we will impute data using the “best-worst-case analysis”, and the “worst-best-case analysis” [35]. For continuous outcomes, we will use available-participant analysis. We will impute the standard deviation from *P* values according to the Cochrane Handbook for Systematic Reviews of Intervention [33]. If it is not possible to calculate the standard deviation from the *P* value or the confidence intervals, we will impute the highest standard deviation in the other trials that included the relevant outcome—we recognise that this will decrease the weight of the trial in our analysis and may bias the effect estimate to no effect.

### Additional analysis

To facilitate interpretability of our results, we will additionally conduct the above analysis with death or serious neonatal morbidity as the outcome.

## 2. Trial sequential analysis

We wish to control the risks of both type I and type II errors. We will therefore perform trial sequential analysis on all primary and secondary outcomes, in order to calculate the required information size (that is, the number of participants needed in a meta-analysis to detect or reject a certain intervention effect) and the cumulative *Z*-curve’s breach of relevant trial sequential monitoring boundaries [42–50]. A detailed description of trial sequential analysis can be found in the Trial Sequential Analysis Manual [43] and at http://www.ctu.dk/tsa/. For dichotomous outcomes, we will estimate the required information size based on the observed proportion of patients with an outcome in the control group (the cumulative proportion of patients with an event in the control groups relative to all patients in the control groups), a relative risk reduction or a relative risk increase of 20%, an alpha of 2% for all our four primary and secondary outcomes, a beta of 10%, and the observed diversity as suggested by the trials in the meta-analysis. For continuous outcomes, we will in the Trial Sequential Analysis use the observed standard deviation (SD), a mean difference equal to the observed SD/2, an alpha of 2% for all outcomes, a beta of 10%, and the observed diversity as suggested by the trials in the meta-analysis.

## 3. Individual participant data meta-analysis

Results of individual patient data meta-analysis may increase the possibility to identify subgroups of patients with specific effects of the assessed interventions. If we receive individual patient data, we will analyse these data using a one-stage analysis model. We will analyse whether there is significant interaction between the intervention variable and ‘trial’ and ultimately decide whether the pooling all available data is warranted. To consider the clustering of participants within trials, we will use generalised estimating equations (GEE). This analysis will be adjusted for the common categoric baseline variables that all the trials used as stratification variables in their randomisation. When analysing continuous data, we will adjust all analyses for the baseline values of the variable (e.g. the outcome of gestational age at birth will be adjusted for baseline values of gestational age at randomisation) [33]. In addition, we will adjust for and perform subgroup analyses for the following potential confounders of the association between early onset growth restriction and perinatal mortality or severe neonatal morbidity, determined at the time of trial inclusion: (1) gestational age; (2) abdominal circumference; (3) estimated fetal weight; (4) umbilical artery Doppler waveform status; (5) uterine artery Doppler waveform status; (6) hypertensive disorder of pregnancy status; (7) abnormal or normal PLGF level, as defined by individual trials; (8) whether or not the trial was a STRIDER trial; (9) daily dose of PDE-5 inhibitor used; (10) type of PDE-5 inhibitor used; (11) trial-level risk of bias assessment; and (12) trial industry sponsorship.

We will secondly conduct a two-stage analysis, where we at the 1st stage will reduce available individual patient data to aggregate data for each trial, and at the 2nd stage will combine all available data in a meta-analysis using the generic inverse variance method, and both fixed-effect and random-effects models.

### Assessments of underlying statistical assumptions

We will systematically assess underlying statistical assumptions for all statistical analyses [51, 52]. In short, for all regression analyses, we will test for major interactions between each covariate and the intervention variable. For each combination, we will test if the interaction term is significant and assess the effect size. We will only consider that there is evidence of an interaction if the interaction is statistically significant after Bonferroni adjusted thresholds (0.05 divided by number of possible interactions) and if the interaction shows a clinically significant effect. If it is concluded that the interaction is significant, we will consider both presenting an analysis separately for each (e.g. for each site if there is significant interaction between the trial intervention and ‘site’) and an overall analysis including the interaction term in the model [51, 52]. For detailed description of the planned assessments for underlying assumptions, please consult the recommendations of Nørskov et al. and Nielsen et al. [51, 52].

### Dealing with missing data

We will use intention to treat data where available. We will deal with missing data according to previously recommended methods [53].

## Discussion

In this pre-planned systematic review with individual participant data meta-analysis, we will examine the effect of PDE-5 inhibitors on the outcome of intact neonatal survival, among pregnancies with fetal growth restriction. These results may provide robust evidence to guide clinical practice and future research regarding the use of this medication for fetal growth restriction.

This detailed statistical analysis plan has a number of strengths. The predefined methodology is based on The Cochrane Handbook for Systematic Reviews of Interventions [33], the eight-step assessment suggested by Jakobsen et al. [35], Trial Sequential Analysis [42] and GRADE [54]. Hence, this protocol considers both risks of random errors and risks of systematic errors. This pre-defined plan will aid in minimising the risk of bias in results and interpretation.

Our protocol also has some limitations. Our study may face practical challenges in terms of obtaining individual participant data from eligible trials outside of the STRIDER Consortium. We will attempt to contact study investigators multiple times, as outlined in our protocol, and we will be as flexible as possible regarding timelines to receive data in order to mitigate potential barriers to collaboration. Second, it may be challenging to merge data from trials conducted outside of the STRIDER Consortium with the STRIDER trials, since study methods or data variables may be different or may not translate to STRIDER variables. Finally, we assess several outcomes and subgroup analyses which increases the risk of type I errors. We plan to use Trial Sequential Analysis to adjust thresholds for significance and we adjust the thresholds for significance according to the number of main outcomes, but we do not take into account the total number of comparisons. This large risk of type 1 error will be considered when interpreting the review results.

This protocol has a few main differences compared to our previously published study protocol [18]. First, we amended the timing of assessment of our primary outcome, which was originally set at “term age”, and will now be to the time of maximum follow-up within 1 year from randomisation. For each included trial we will report the timing at which assessments were made which may include ‘term age’, ‘primary hospital discharge’, or specific infant ages < 12 months or time from randomisation. Second, we amended our risk of bias assessment to use the updated Cochrane RoB2 tool [28], which was not published when the previous protocol was developed. Third, we amended our subgroup analyses to add relevant comparisons, such as those based on gestational age at inclusion. Finally, we amended our analysis plan to include both a one-stage and two-stage approach to optimise assessment of variables of interest (since the one-stage model only allows for adjustment of variables that were uniformly assessed in all trials), and to provide further information about the replicability and validity of our findings [33]. Any further amendments made to this protocol when conducting the study will be outlined and reported in the final manuscript.

We will disseminate results from this study through a peer-reviewed publication. We will also share results at national and international conferences, and through communications disseminated by national and international professional organisations such as the Society of Maternal Fetal Medicine, the Perinatal Society of Australia and New Zealand (PSANZ), the British Maternal Fetal Medicine Society (BMFMS), and the Society of Obstetricians and Gynaecologists of Canada (SOGC). We will also incorporate study findings into national clinical practice guidelines which our team members are involved in.

## Supplementary Information

**Additional file 1:.** PRISMA-IPD checklist of items to include when reporting a systematic review and meta-analysis of individual participant data (IPD)

**Additional file 2:.** Draft MEDLINE search

**Additional file 3:.** Data variables

## Data Availability

Not applicable.

## Abbreviations

PDE-5: Phosphodiesterase type 5
STRIDER: Sildenafil TheRapy In Dismal prognosis Early-onset fetal growth Restriction [Consortium]
PLGF: Placental growth factor
