# Supplementary material for: Short-term outcomes of phosphodiesterase type 5 inhibitors for fetal growth restriction: a study protocol for a systematic review with individual participant data meta-analysis, aggregate meta-analysis, and trial sequential analysis
Source: Syst Rev. 2021 Dec 3;10:305. doi: 10.1186/s13643-021-01849-5 (PMC8643016; doi:10.1186/s13643-021-01849-5)
Supplement: Supplementary file 2 — Additional file 2:. Draft MEDLINE search [file 13643_2021_1849_MOESM2_ESM.docx]

**Additional File 2: Draft MEDLINE Search**

Database(s): **Ovid MEDLINE(R) and Epub Ahead of Print, In-Process & Other Non-Indexed Citations and Daily** 1946 to September 11, 2020
Search Strategy: **2020-09-13**

| # | Searches | Results |
| --- | --- | --- |
| 1 | fetal growth retardation/ | 16397 |
| 2 | placental insufficiency/ | 1647 |
| 3 | exp hypertension, pregnancy-induced/ | 36774 |
| 4 | ((f?etal or f?etus* or intrauterin* or uterine or utero or antenat* or prenat* or early-onset or antenat* or ante-nat* or prenat* or pre-nat* or first trimester or 1st trimester) adj6 (growth adj3 (retard* or restrict*))).tw,kf. | 20771 |
| 5 | (FGR* or IUGR*).tw,kf. | 7752 |
| 6 | ((early or pregnancy or gestational) adj2 (growth retard* or growth restrict*)).tw,kf. | 695 |
| 7 | ((estimat* adj4 (f?etal or f?etus*) adj2 weight*) or EFW).tw,kf. | 2201 |
| 8 | (((absent or revers*) adj6 (enddiastol or diastol* or doppler) adj2 (velocity or flow)) or AEDF* or A-EDF* or AREDF* or A-REDF*).tw,kf. | 1100 |
| 9 | (abdom* adj3 circumfer*).tw,kf. | 3430 |
| 10 | (placent* adj3 (insufficien* or d#sfunct* or disorder*)).tw,kf. | 3925 |
| 11 | (((gestational or maternal or pregnancy) adj (hypertension or high blood pressur*)) or pregnancy-induced hypertension or (pregnancy adj3 hypertensive disorder*)).tw,kf. | 9805 |
| 12 | (HELPP or preeclam* or eclam*).tw,kf. | 36908 |
| 13 | **or/1-12 [ FGR, placental insufficiency, pregnancy hypertensive disorders ]** | **83181** |
| 14 | phosphodiesterase 5 inhibitors/ or sildenafil citrate/ or tadalafil/ | 7908 |
| 15 | (sildenafil* or revatio or homosildenafil or hydroxyhomosildenafil or Viagra or acetildenafil or desmethylsildenafil or NCX-911 or NCX911 or UK-92480* or UK92480* or tadalafil* or cialis or ic-351 or ic351).tw,kf. | 8454 |
| 16 | (((((phosphodiesteras* or phospho-diesteras* or PDE) adj3 ("5" or V)) or phosphodiesterase5 or PDE5 or phosphodiesteraseV or PDEV) adj5 (inhib* or block* or antagonist* or anti)) or PD5i* or PD5-I or PDVi* or PDV-i).tw,kf. | 6289 |
| 17 | **or/14-16 [PD5-inhibitors]** | **12976** |
| 18 | **13 and 17 [ FGR & PD5-inhibitors ]** | **139** |
| 19 | (TADAFER or (Strider not (short-tandem-repeat or DNA-strider* or ((water or oil or bike or bikes or biped*) adj5 strider*)))).ti. | 15 |
| 20 | **18 or 19 [ FGR & PD5-inhibitors -Strider/Tadafer trial ]** | **140** |
| 21 | ((controlled clinical trial or randomized controlled trial).pt. or double-blind method/ or placebos/ or (random* or (controlled adj3 (study or trial)) or placebo* or double-blind*).tw,kf. or trial.ti.) not (("systematic review" or review).pt. or review.ti.) [ RCT-filter adapted from the Cochrane, reviews excluded ] | 1336887 |
| 22 | (exp animals/ not humans/) or ((pig or pigs or goat or goats* or sheep or lamb or lambs or ovine or rodent* or rabbit* or mice or mouse or murine* or rat or rats).ti. not human*.ti,ot.) [ animal filter] | 4966334 |
| 23 | **21 not 22 [ human RCT filter, adapted from the Cochrane ]** | **1189300** |
| 24 | **20 and 23 [ human RCTs on FGR & PD5-inhibitors ]** | **33** |
| 25 | **remove duplicates from 24 [ human RCTs on FGR & PD5-inhibitors, deduplicated ]** | **33** |
