# Supplementary material for: Short-term outcomes of phosphodiesterase type 5 inhibitors for fetal growth restriction: a study protocol for a systematic review with individual participant data meta-analysis, aggregate meta-analysis, and trial sequential analysis
Source: Syst Rev. 2021 Dec 3;10:305. doi: 10.1186/s13643-021-01849-5 (PMC8643016; doi:10.1186/s13643-021-01849-5)
Supplement: Supplementary file 3 — Additional file 3:. Data variables [file 13643_2021_1849_MOESM3_ESM.docx]

| Category | Data variables |
| --- | --- |
| Population | Maternal characteristics at study entry   - Age. - Body mass index. - At least one previous pregnancy ≥ 20 weeks. - Ethnicity. - Smoking status. - Pre-existing hypertension requiring medication. - Diabetes (type 1, type 2, and gestational). - Gestational hypertension. - Preeclampsia. - Gestational age at diagnosis of preeclampsia. - Concurrent treatment with   - acetylsalicylic acid (e.g. Aspirin).   - low molecular weight heparin.   Pregnancy characteristics at trial entry   - Gestational age (based on ultrasound assessment at < 14 weeks gestation):   - < 24 weeks + 0 days   - ≥ 24 weeks + 0 days   - Gestational age, weeks. - Abdominal circumference centile.^26^ - Estimated fetal weight, g (Hadlock C).^50^ - Umbilical artery:   - Mean pulsatility index.   - End diastolic flow absent or reversed.   - Mean pulsatility index ≤ 95^th^ centile (normal).   - Mean pulsatility index > 95^th^ centile (abnormal). - Mean pulsatility index of middle cerebral artery. - Uterine artery:   - Mean pulsatility index.   - Unilateral or bilateral notching.   Pregnancy characteristics after trial entry   - Development of preeclampsia and gestational age at diagnosis of preeclampsia. - Antenatal corticosteroids administered for fetal lung maturity. - Magnesium sulphate administered. |
| Intervention | - Type of PDE-5 inhibitor - Daily dose of PDE-5 inhibitor - Number of days PDE-5 inhibitor was administered |
| Comparison | - Placebo - No treatment |
| Outcome | Primary outcome  Serious adverse neonatal outcome, defined as one or more of the following:   - Cerebral intraventricular haemorrhage (Papile grade three or four, or as defined by individual trials) - Cystic periventricular leukomalacia (grade two or more, or as defined by individual trials). - Bronchopulmonary dysplasia (as defined by individual trials). - Necrotising enterocolitis requiring surgery (as defined by individual trials). - Retinopathy of prematurity requiring treatment (as defined by individual trials).   **Secondary outcomes**  *For the neonate*   - Gestational age at birth (among liveborn neonates). - Birth weight z-score (among liveborn neonates).   *For the woman*   - Maternal preeclampsia (as defined by individual trials).   **Exploratory outcomes**  *For the neonate*   - Persistent pulmonary hypertension in the neonate (as defined by individual trials). - Stillbirth. - Neonatal or infant death. - Cerebral intraventricular haemorrhage (Papile grade three or four or as defined by individuals trials). - Cystic periventricular leukomalacia (grade two or more, or as defined by individual trials). - Bronchopulmonary dysplasia (as defined by individual trials). - Necrotising enterocolitis requiring surgery (as defined by individual trials). - Retinopathy of prematurity requiring treatment (as defined by individual trials). - Persistent pulmonary hypertension as defined by the Consortium (after completion of the trials). - Use of nitric oxide (neonatal). - Fetal growth velocity post-treatment:   - Post-treatment growth velocity will be calculated from Z-scores at the recruitment and day 14 assessments. Where delivery or fetal death occurred before the day 14 assessment, the longest interval available will be used (i.e. to a minimum of 48 hours). - Birthweight (grams). - Pregnancy prolongation (in days).   *For the woman*   - Maternal systolic blood pressure 48-72 hours after commencing treatment. - Maternal diastolic blood pressure 48-72 hours after commencing treatment. - Mode of birth: vaginal compared to caesarean section. - Abnormal maternal serum placental growth factor (PLGF) (as defined by individual trials). |
